# Genetic and molecular characterization of metabolic pathway-based clusters in esophageal squamous cell carcinoma

**DOI:** 10.1038/s41598-024-56391-w

**Published:** 2024-03-14

**Authors:** Ze Wang, Yuan Zhang, Xiaorong Yang, Tongchao Zhang, Zhen Li, Yang Zhong, Yuan Fang, Wei Chong, Hao Chen, Ming Lu

**Affiliations:** 1 Clinical Epidemiology Unit, Clinical Research Center of Shandong University, Qilu Hospital of Shandong University, Jinan, 250012 Shandong China; 2 Department of Epidemiology and Health Statistics, School of Public Health, Cheeloo College of Medicine, Shandong University, Jinan, 250012 Shandong China; 3 Department of Gastrointestinal Surgery, Key Laboratory of Engineering of Shandong Province, Shandong Provincial Hospital Affiliated to Shandong First Medical University, Medical Science and Technology Innovation Center, Shandong First Medical University & Shandong Academy of Medical Sciences, Jinan, China

**Keywords:** Cancer metabolism, Tumour heterogeneity, Oesophageal cancer

## Abstract

Esophageal squamous cell carcinoma (ESCC) is one of the most aggressive types of squamous cell carcinoma and represents a significant proportion of esophageal cancer. Metabolic reprogramming plays a key role in the occurrence and development of ESCC. Unsupervised clustering analysis was employed to stratify ESCC samples into three clusters: MPC1-lipid type, MPC2-amino acid type, and MPC3-energy type, based on the enrichment scores of metabolic pathways extracted from the Reactome database. The MPC3 cluster exhibited characteristics of energy metabolism, with heightened glycolysis, cofactors, and nucleotide metabolism, showing a trend toward increased aggressiveness and poorer survival rates. On the other hand, MPC1 and MPC2 primarily involved lipid and amino acid metabolism, respectively. In addition, liquid chromatography‒mass spectrometry-based metabolite profiles and potential therapeutic agents were explored and compared among ESCC cell lines with different MPCs. MPC3 amplified energy metabolism markers, especially carnitines. In contrast, MPC1 and MPC2 predominantly had elevated levels of lipids (primarily triacylglycerol) and amino acids, respectively. Furthermore, MPC3 demonstrated a suboptimal clinical response to PD-L1 immunotherapy but showed increased sensitivity to the doramapimod chemotherapy regimen, as evident from drug sensitivity evaluations. These insights pave the way for a more personalized therapeutic approach, potentially enhancing treatment precision for ESCC patients.

## Introduction

Esophageal squamous cell carcinoma (ESCC) and esophageal adenocarcinoma (EAC) are two histological subtypes of esophageal cancer (EC) but exhibit significant differences at the molecular and pathological levels^1^. ESCCs represent approximately 90% of all ECs, and Eastern to Central Asia has a particularly heavy disease burden^2,3^. However, the prognosis of ESCC patients is poor, and the overall 5-year survival rate is low. Even with the advances of surgery, chemotherapy, and radiotherapy, the 5-year overall survival rate is only 15.3% in advanced stages^4^. Therefore, achieving the goal of effective treatment remains challenging.

Metabolic reprogramming is crucial to tumor initiation, progression, and metastasis^5,6^. Cancer cells need to increase glucose uptake and fermentation of glucose to lactate to meet the needs of growth, survival, proliferation, and long-term maintenance^7^. Moreover, heterogeneity is evident between cancers from different patients^8^. This phenotypic diversity, marked by distinct cell surface markers, genetic abnormalities, and other cancer hallmarks, can influence prognostic variations and lead to failures in uniform treatments^9^. Tumor individuals have highly heterogeneous metabolic profiles, and precise classification enables the identification of sophisticated targeted therapies to enhance their efficacy^10^.

Much effort has been devoted over the past decade to classifying ESCC into several molecular subtypes. Moreover, distinct mutational profiles, genomic alterations and biological process stratifications could guide treatment decisions, with the prognosis improved^11–14^. Moreover, there is growing appreciation that circulating metabolite dysregulation is frequently observed in ESCC patients^15,16^. In the metabolic process of ESCC, a number of metabolites are differentially distributed and involved in glycolysis, anaerobic respiration, the tricarboxylic acid cycle, and protein and lipid metabolism^17^. Lactic acid, citrate, glucose and valine are the main reported metabolites involved in modulating ESCC progression and prognosis.

However, there is a lack of studies focusing on metabolic pathway-based stratification, which could provide insight into the metabolic dysregulation and inherent heterogeneity in ESCC. Given that metabolic reprogramming is a recognized hallmark of cancer, pinpointing the metabolic status of tumors holds utmost significance. Our study performed a comprehensive metabolic transcriptional clustering analysis of ESCCs based on metabolic pathway profiles. We successfully identified three distinct clusters, each correlated with specific clinical characteristics and metabolic pathways. Our analysis showcased the metabolic heterogeneity of ESCC tissues, laying the foundation for the development of personalized therapies customized to individual tumor metabolic profiles.

## Methods

### Transcriptomic expression and clinical data

The transcriptomic expression profiles of ESCC patients in the TCGA-ESCA cohort (The Cancer Genome Atlas-Esophageal Carcinoma, N = 182) were obtained from the cBioPortal (https://www.cbioportal.org/). Clinical information, including age, sex, clinical stage, TNM stage, and survival time, was also retrieved. Patients categorized as EAC type, of unknown type, or without survival data were omitted. This filtering resulted in 90 patients being selected for our study. cBioPortal preprocessed these data, including quality control, data cleaning, standardization, to ensure the accuracy and comparability of the data. During RNA-Seq processes in ESCC tissues or the conversion of raw data to RSEM format in cBioPortal, certain genes or transcripts may have incomplete or unavailable sequencing data due to technological limitations. The missing values of the RNA-seq data were imputed by using the knnimputation function of the R package ‘DMwR2’ (v.0.0.2).

The other two ESCC datasets (GSE53625, N = 358 and GSE121931, N = 125) were procured from the NCBI Gene Expression Omnibus (GEO) database (https://www.ncbi.nlm.nih.gov/geo/). The GSE53625 dataset was utilized for exploratory analysis because it met our criteria: (1) contained human esophageal tissue samples, (2) contained at least 100 samples, (3) provided gene symbol annotations, and (4) had prognostic information. This cohort, consisting of 179 ESCC samples, was the most extensive dataset in our study, establishing it as the primary dataset for discovery and analyses. The GSE121931 dataset was another GEO dataset comprising 125 patients, specifically chosen to validate our metabolic pathway stratification. The raw data of the two GEO datasets were processed by the robust multiarray averaging (RMA) algorithm with ‘affy’ package and the duplicate probes were merged via median number.

### Metabolic pathway-based classification

To investigate the metabolic heterogeneity in ESCC, we manually selected 82 metabolic pathways (Table S1) from the Reactome Pathway Database (https://reactome.org/). We used gene set variation analysis (GSVA) with the R package 'GSVA' (v.1.42.0) to calculate the enrichment scores of metabolic pathways in each sample. The relevance of the pathways was explored with Pearson correlation. To investigate the metabolic heterogeneity within the ESCC tumors, the unsupervised clustering nonnegative matrix factorization (NMF) method was utilized for consensus clustering of metabolic pathway-based clusters (MPCs). The R packages ‘NMF’ (v.0.23.0) and ‘doMPI’ (v.0.2.2) with the Lee algorithm were adopted to stratify the samples. The optimal number of clusters was determined according to cophenetics, consensus matrices and silhouette coefficients. The most suitable rank value was determined by identifying the point where the cophenetic value exhibited the most significant change in response to variations in the cluster count. To enhance the validity of our judgments, we employed supplementary tools such as heatmaps. These visual aids provided a clear snapshot of the data, enabling a more precise evaluation. 84 metabolic pathways extracted from the Kyoto Encyclopedia of Genes and Genomes (KEGG) database (http://www.kegg.jp/) were used to validate cluster characteristics (Table S2).

### PPI network analysis of the integrated DEGs

We identified differentially expressed genes (DEGs) related to metabolism by pairwise comparisons of clusters using the 'limma' R package (v.3.50.3) and considered the union set of these comparison results as the DEGs among the three MPCs^18^. The metabolic-related DEGs were further filtered by intersection with DEGs between tumor and normal tissues. The normalized gene expression data were subsequently analyzed with the lmFit and eBayes functions to calculate expression statistics. The significance criterion for DEGs was a *false discovery rate* (*FDR*) less than 0.001.

The Search Tool for the Retrieval of Interacting Genes/Proteins database (STRING, https://string-db.org/) was used to investigate and visualize the gene interactions. The DEGs obtained above were submitted to the STRING database to determine their protein‒protein interactions (PPIs). We imported the PPIs into Cytoscape software (v.3.9.0) to construct a network and exclude nodes without betweenness. The PPI network ultimately contained 174 filtered DEGs (Table S3). Gene Ontology (GO) enrichment analysis was used to explore the potential biological processes, cell compositions and molecular functions of the filtered DEGs with the R package ‘clusterProfiler’ (v.4.2.2)^19^.

### Genomic mutations and mutational signature analysis

Mutation and copy number alteration (CNA) data in mutation annotation format (MAF) from the TCGA cohort were also obtained from the cBioPortal. Mutational landscape depiction and signature extraction were both applied in the ‘maftools’ package. The ExtractSignatures function based on Bayesian variant nonnegative matrix factorization factorizes the mutation portrait matrix into two nonnegative matrices, ‘signatures’ and ‘contributions’, where ‘signatures’ represent mutational processes and ‘contributions’ represent the corresponding mutational activities^20^. The SignatureEnrichment function automatically determines the optimal number of extracted mutational signatures and assigns them to each sample based on mutational activity. The extracted mutational portraits of CRC were compared and annotated by cosine similarity analysis against the Catalog of Somatic Mutations in Cancer (COSMIC) database.

### Immune cell infiltration analysis

CIBERSORT (http://cibersort.stanford.edu/), a deconvolution algorithm based on transcriptome data, was used to estimate the composition and abundance of immune cells in complex tissues. The method provided a known dataset of gene expression features for 22 immune cell subsets downloaded from the study by Ali et al.^21^. xCell (https://xcell.ucsf.edu/) is another computational method based on single-sample GSEA (ssGSEA). The method transforms gene expression profiles into enriched fractions of 64 immune and stromal cell types across samples. The relative abundance of each immune cell type was represented by an enrichment score. The two methods were combined to explore differences in immune infiltration among MPCs.

We also used the R package ‘IOBR’ (v.0.99.9)^22^ to analyze the whole landscape of immune cell signatures. The package integrates eight published methods for quantifying the percentage of signatures and gathers the results for all methods. The ssGSEA method was also used for our analysis. In addition to analyzing immune signatures, this package is equipped to analyze the tumor environment, m6A status, metabolism, exosomes, and microsatellite instability (MSI) status, providing a comprehensive overview of the results.

### Functional enrichment analysis

The GSVA method was also used to explore the biological pathways extracted from the RNA-seq data. The Hallmark gene sets, which represent clearly defined biological states and processes, were obtained from the Molecular Signatures Database (MSigDB, http://www.gsea-msigdb.org/gsea/msigdb/collections.jsp). Gene set enrichment analysis (GSEA) was also applied to identify the gene sets that were significantly enriched among the MPCs. The R package ‘limma’^18^ was used to evaluate the differential expression of more than 20,000 genes in samples from different groups. Genes sorted according to logFC values produced by limma were used as inputs for GSEA with the R package ‘clusterProfiler’ (v.4.2.2)^19^ against the KEGG pathways; these data were also downloaded from the MSigDB as reference gene sets.

### Metabolite profile analysis

We utilized cell lines to explore the relative levels of metabolites in the Cancer Cell Line Encyclopedia (CCLE) database. The metabolome landscape of esophageal cancer cell lines was collected from Li et al. study^23^. We selected 27 esophageal cancer cell lines with ESCC annotations. The transcriptome data were downloaded from the Dependency Map (DepMap) portal. The cell lines were stratified by the NMF algorithm using the same analysis pipelines used in this study. A total of 225 metabolites profiled by liquid chromatography–mass spectrometry (LC–MS) were retained in 26 available cell lines. Finally, we compared the metabolite levels among the three MPCs.

### Drug sensitivity analysis

We used the R package ‘oncoPredict’ (v.0.2)^24^ to construct the drug sensitivity prediction procedure. The imputations were performed based on the expression matrix of a training set with known drug treatment information against the Genomics of Drug Sensitivity in Cancer (GDSC) database. The phenotypes (drug sensitivity scores) of the samples were calculated using ridge regression with bulk RNA-seq data.

### Immunotherapy cohort analysis

An immunotherapeutic cohort of patients with advanced squamous cancer^25^ treated with atezolizumab (anti-PD-L1 mAb) was utilized to further explore the immunotherapeutic outcomes based on our NMF classification method. The transcriptome profiles and detailed clinical annotations with immune response information were downloaded from the R package ‘IMvigor210CoreBiologies’ (v.1.0.0), with 295 patients enrolled in our analysis. We acquired enrichment scores based on metabolic pathway enrichment via the GSVA method. The samples were also classified as MPCs by NMF using the Lee algorithm based on enrichment scores. Given the association between tumor neoantigen burden and immunotherapy efficacy, we retained a total of 214 samples that contained neoantigen burden information to probe differences among the clusters. Each MPC subtype was subsequently segmented into high- and low- neoantigen burden groups based on the median count and labeled HMPC or LMPC, respectively.

### Statistical analysis

Statistical analysis was performed using R software (v.4.1.3). For normally distributed variables, Student’s t test and one-way ANOVA were separately used to perform the group comparisons of two groups and more than two groups, respectively. Otherwise, the Wilcoxon rank-sum test or Kruskal‒Wallis test was used as a nonparametric test. The χ^2^ test or Fisher’s exact test was used to analyze categorical variables. K‒M survival analysis and a Cox proportional hazards model were used to analyze the association between the MPCs and patient prognosis with the R packages ‘survival’ (v. 3.2.13) and ‘survminer’ (v.0.4.9). Cox multivariate regression was performed with the R package ‘ezcox’ (v.1.0.2). Unless otherwise mentioned, *P* < 0.05 was considered to indicate statistical significance.

## Results

### Three distinct MPCs in ESCC tissues based on NMF analysis

**Figure 1:**
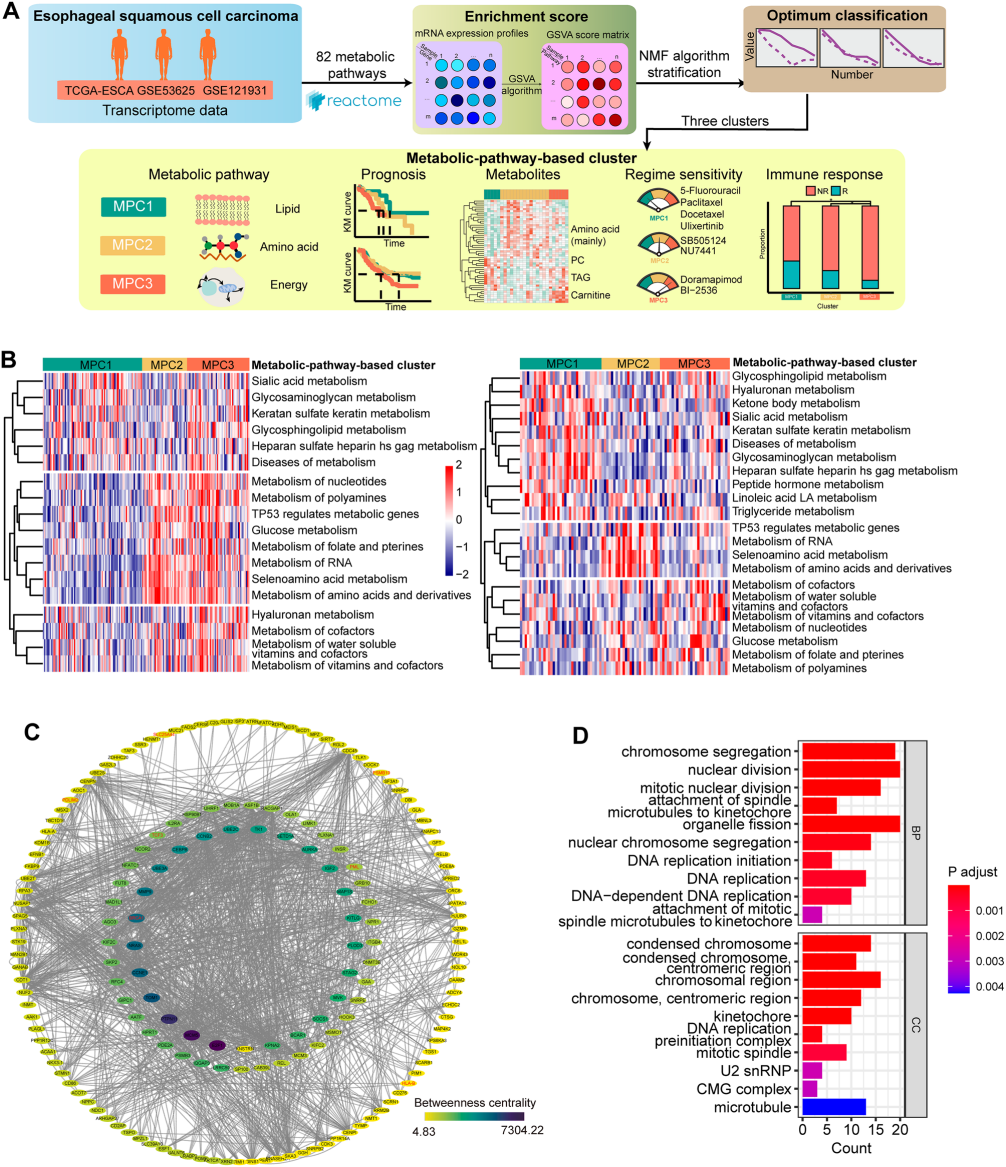
Metabolic pathway-based clustering results and identification of genes differentially expressed with respect to tumor metabolism in ESCC samples. (**A**) Workflow of metabolic pathway-based stratification. (**B**) Heatmap showing normalized enrichment scores of the three MPCs in the TCGA and GSE53625 cohorts. The significant metabolic pathway enrichment scores acquired from both datasets are plotted. (**C**) Construction of a protein‒protein interaction (PPI) network with 174 filtered DEGs. The size and color of nodes were associated with betweenness. Annotations in red indicate prognosis-related genes. (**D**) Dot plot presenting the results of Gene Ontology (GO) functional enrichment analysis of 174 filtered DEGs in the PPI network.

Previous studies have demonstrated heterogeneity in metabolic gene expression within tumors and across different cancer types^26^. In this study, we aimed to investigate this heterogeneity using transcriptomic data. The overall workflow of the metabolic stratification is shown in Fig. 1A. Correlation analysis of the 82 pathway enrichment scores identified clusters of pathways that were either coactivated or corepressed across the dataset (Fig. S1A). The gene set enrichment scores of the extracted metabolic pathways were calculated with the GSVA algorithm for each sample. After conducting an NMF rank survey analysis with group numbers K ranging from two to seven, we determined that the optimal clustering number was three. The consensus matrices of different ranks in the three datasets and the mixture coefficients of rank three are illustrated in the heatmap (Fig. S1B–E). This determination arose from the significant decrease in the cophenetic correlation coefficient observed when the clustering number was set to three. This dip indicated a more distinct and meaningful clustering pattern compared to other values of K, thus suggesting that three clusters best represented the underlying structure in the data (Fig. S2). Additional NMF rank survey indices supported our classification approach. The silhouette analysis further confirmed the appropriateness of our clustering configuration by revealing a high value for the objects assigned to cluster three. We integrated the distinct pathways from the two exploration datasets (GSE53625 and TCGA) to characterize the metabolic profiles of the different MPCs (labeled MPC1, MPC2, and MPC3), and verified the characteristic identification using the validation dataset (GSE121931). Overall, the relative scores of metabolic pathways among the MPCs exhibited unique patterns (Figs. 1B, S3A). MPC1 can be classified as a lipid type and includes glycosphingolipid, heparin, triglyceride and linoleic acid. MPC2 was characterized by the upregulation of amino acid metabolism, especially that of selenoamino acids. MPC3 was identified as an energy metabolism cluster that upregulates glucose metabolism and nucleotide metabolism, with concurrent upregulation of vitamin and cofactor metabolism. To further validate the metabolic profiles of our clusters, we reanalyzed the enrichment score among clusters using the KEGG database, and found that the metabolic pathways with statistically significant differences of MPCs were consistent with the results via Reactome database (Fig. S3B).

### The PPI network helps identify significant DEGs associated with tumor metabolism

To explore the impact of metabolic heterogeneity and differences between tumor and normal tissues, we conducted an interaction analysis of DEGs. We identified a total of 2696 DEGs between tumor and normal samples. In addition, 4371 DEGs were found to be related to metabolic pathways and had a false discovery rate (FDR) of less than 0.001. The DEGs mentioned above were all obtained by intersecting data from the GSE53625 and TCGA cohorts.

We subsequently overlapped the two sets of DEGs, culminating in a consolidated list of 368 genes. After removing isolated nodes and genes with zero betweenness, 174 genes were identified and utilized to construct a protein‒protein interaction (PPI) network (Table S3). E2F1 was the gene with the most pronounced difference in betweenness centrality, highlighting its critical role within the network (Fig. 1C). Notably, E2F1 exhibited higher expression levels of MPC3 and MPC2 than did MPC1, suggesting its potential role in the metabolic heterogeneity observed among the metabolic pathway clusters. Furthermore, we identified seven genes that showed prognostic significance, namely, BCAR1, HLA-B, PDLIM2, PML, PSMB10, SLC25A44 and TCF3 (Fig. S3C). We also analyzed the association between survival and lncRNAs in the GSE53625 dataset using univariate Cox regression. A total of 162 lncRNAs were significantly associated with overall survival (OS) (Table S4 and Fig. S3D). Additionally, we observed differential expression of two negatively correlated prognostic lncRNAs (LINC01867 and CTBP1-AS) and four positively correlated prognostic lncRNAs (TTTY3, TPM1-AS, LINC01132, and VIM-AS1) among the MPCs (Fig. S3E).

To further investigate the biological function of the combined 174 DEGs in the PPI network, we performed GO pathway enrichment analysis. The results suggested that the DEGs were mainly enriched in nucleotide and ubiquitination metabolic processes with changes in DNA replication processes and chromosomal regions (Fig. 1D), indicating that energy metabolism plays a part in the source of genome instability and is a feature of cancerous cells. Collectively, these results suggested that abnormal gene expression relevant to metabolism in tumors might promote tumor heterogeneity.

### Comparison of clinical features in patients with MPC

**Figure 2:**
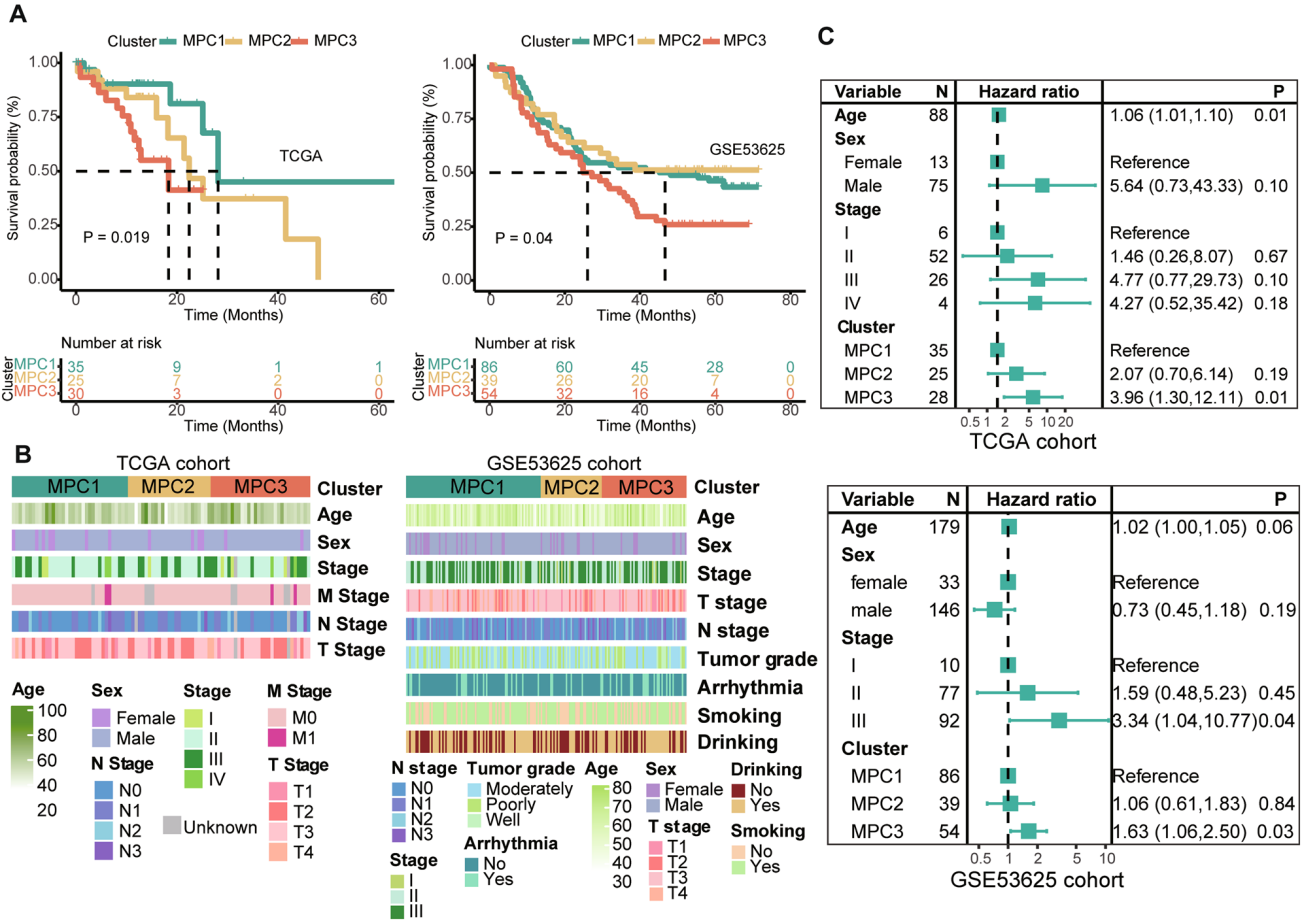
Comparison of clinical features across metabolic pathway-based clusters (**A**) K‒M curves of overall survival (OS) in the three clusters. (**B**) Clinical annotations of the MPCs. (**C**) Forest plot of the hazard ratio (HR) determined by multivariate Cox regression after adjusting for age, sex and tumor stage showing the prognostic value of the MPCs.

The three MPCs also exhibited different clinical characteristics. These genes were associated with OS in both the TCGA (log-rank test *P* = 0.019) and GSE53625 (log-rank test *P* = 0.039) cohorts (Fig. 2A). Notably, patients in the MPC3 exhibited poorer OS than patients in the other two clusters did. The clinical annotations and comprehensive statistical descriptions are shown in Figs. 2B and S3F, respectively. For MPC1, the middle section was the predominant location for cancer cells, whereas the upper part also contributed significantly to MPC2 and MPC3 expression. Univariate Cox regression showed that tumor stage and MPC were significantly different between the two cohorts (Fig. S3G). Multivariate Cox regression also revealed that MPC3 predicted a worse survival outcome after adjusting for age, sex, and TNM stage, with MPC1 used as a reference (*P* = 0.01 and 0.03, respectively; Fig. 2C). Associated with the characteristics of MPCs, tumors with higher rates of energy metabolism dysregulation may be more aggressive than tumors with a high level of lipids and amino acids in ESCC.

### Association of MPCs with tumor genomic alteration profiles

**Figure 3:**
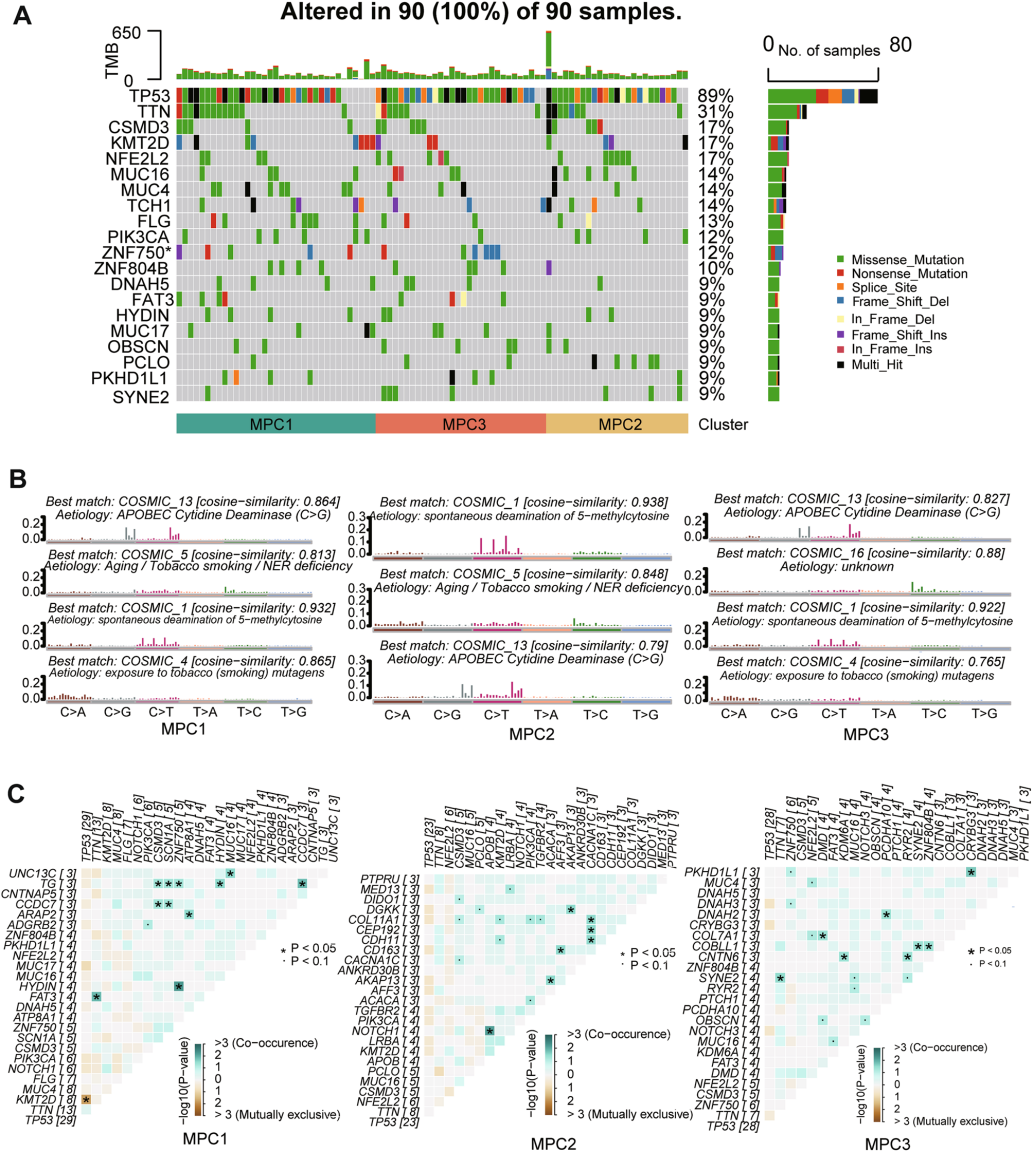
Mutational landscape among metabolic pathway-based clusters of ESCC. (**A**) Oncoplot depicting the distribution of somatic mutation (SNV/indel) and copy number variation (CNV) events affecting frequently mutated genes in ESCC among the MPCs. (**B**) Annotations of curated mutational signatures across MPCs. (**C**) Somatic interactions of the top mutated genes in the MPCs. * means *P* < 0.05.

We investigated the somatic mutational profile and compared the mutational frequency among the three MPCs in the TCGA dataset (Fig. S4A,B). We found that the base mutations consisted largely of C > A and were associated with transition (Ti) > transversion (Tv) in our patients. The waterfall plot showed that TP53 was the most common mutational gene (accounting for 89%) in ESCC (Fig. 3A), which was consistent with the findings of previous studies^27^. Among the top 20 mutated genes, ZNF750 was differentially expressed among the three MPCs and had the highest mutation rate in MPC3 (Fisher’s exact test, *P* = 0.048). These findings are consistent with the notion that multifaceted oncogenic regulation of energy metabolism is associated with increased numbers of mutations and has enormous biological significance.

We further investigated the mutational process in ESCC by extracting mutational signatures. The cophenetic metric indicated that the total mutation profiles matched four mutational signatures and were best matched to COSMIC_13, COSMIC_16, COSMIC_1, and COSMIC_4 from the COSMIC database against cosine similarity (Fig. S4C–E). These signatures were annotated as APOBEC cytidine deaminase, drinking, spontaneous deamination of 5-methylcytosine and exposure to tobacco (smoking) mutagens (Fig. S4F). Nevertheless, upon conducting mutant signature enrichment analysis across different MPC groups, we observed some inconsistencies. Specifically, COMSIC_16 was exclusively enriched in MPC3, whereas COMSIC_5 (potentially linked to aging, tobacco smoking, or nucleotide excision repair deficiency) was enriched in both MPC1 and MPC2 (Figs. S4G, 3B).

We identified 11, 7, and 8 cooccurring mutated gene pairs in the MPC1, MPC2 and MPC3 (Fig. 3C). Only one mutually exclusive mutated gene pair (KMT2D and TP53) was found in MPC1. Moreover, ZNF750 mutations cooccured with those of TG and TF in MPC1. Moreover, in MPC3, ZNF750 mutations coincided with mutations in PKHD1L1 and DNAH3 (*P* < 0.01). These findings enabled us to depict the effect of metabolic cluster stratification on genomic variation more comprehensively and to reveal the potentially complex interactions between individual somatic mutations and metabolic heterogeneity.

### Characterization of the immune landscapes of MPCs in ESCC

**Figure 4:**
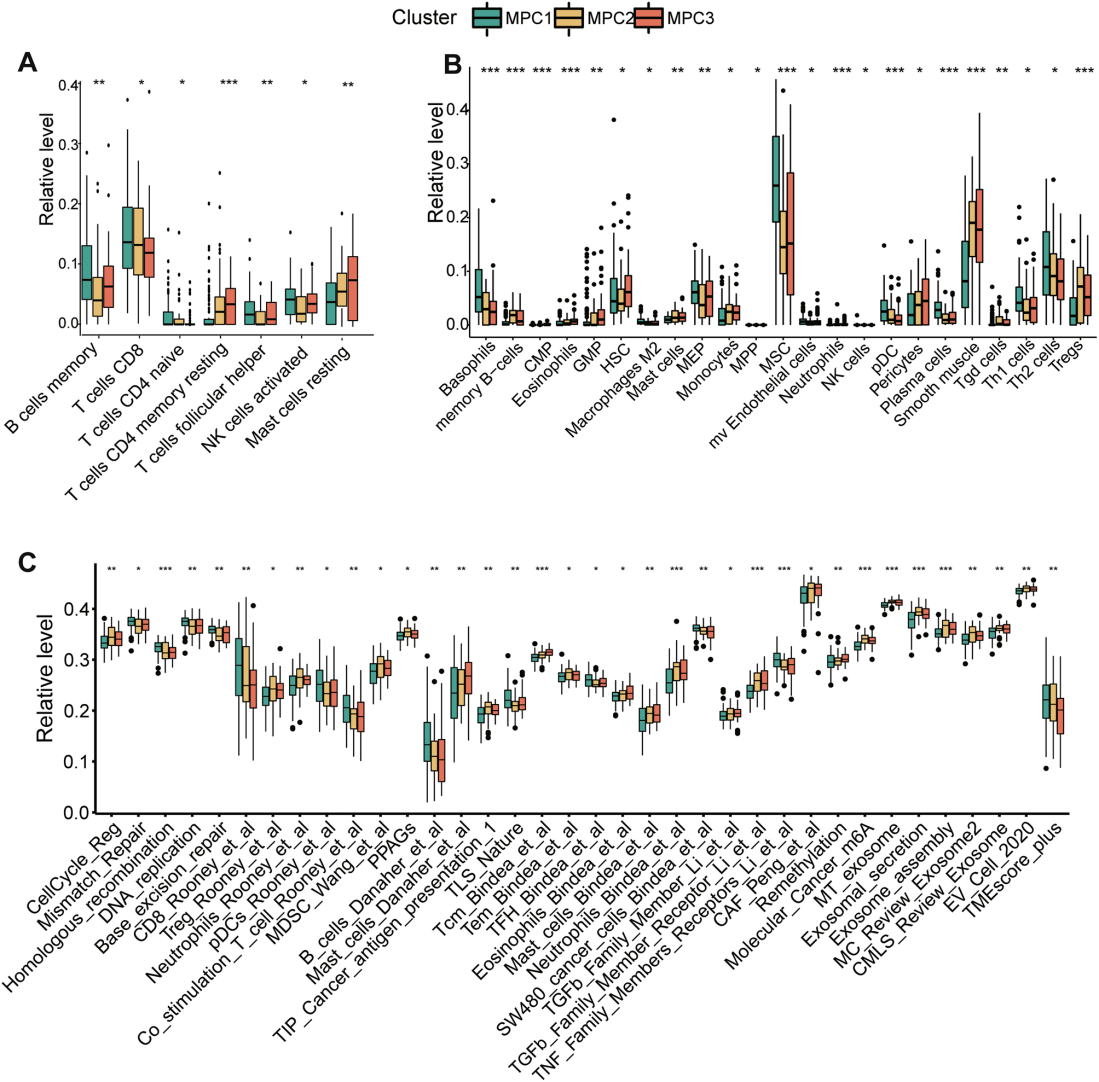
Immune cell and signature landscape among metabolic pathway-based clusters in ESCC patients. The fraction of tumor-infiltrating immune cells in the three clusters determined using the CIBERSORT (**A**) and xCell (**B**) algorithms. (**C**) Comparison of IOBR-based inferred immune signature infiltration levels based on bulk RNA-seq data. Within each group, the scattered dots represent TME cell expression values. The bottom and top of the boxes are the 25th and 75th percentiles (interquartile ranges), respectively. * means *P* < 0.05; ** means *P* < 0.01; *** means *P* < 0.001.

We utilized the CIBERSORT algorithm to characterize immune cell infiltration in the normalized expression matrices of the ESCC dataset (GSE53625 and GSE121931). The proportions of 22 immune cells in ESCC are shown in Fig. S5A. The infiltration of seven types of immune cells significantly differed across the clusters (Figs. 4A, S5B). Changes in resting CD4 memory T cells and resting mast cells were particularly pronounced in MPC3. Two types of immune cells, CD4 naïve T cells and CD8 T cells, were less abundant in the MPC3 population. The remaining four types were highly enriched in MPC1. We further examined differences in immune infiltration with the xCell method. There were notable differences in the presence of mesenchymal stem cells (MSCs) and smooth muscle across the MPCs. Specifically, MSCs were abundant in MPC1, while smooth muscle was notably diminished in this cluster (Fig. 4B). Several other cell types, such as granulocyte monocyte progenitors (GMPs), hematopoietic stem cells (HSCs) and pericytes, were also enriched in MPC3. The M2 macrophage in the MPC3 was greater than that in the other clusters in both GEO datasets (Fig. S5C).

We further compared comprehensive oncology-immune signatures derived from the IOBR package among the three MPCs (Figs. 4C, S5D). Genomic instability-related biological processes, such as mismatch repair, homologous recombination, and DNA replication, were found to be highly expressed in MPC1. The abundances of CD8+ T cells, Tregs and mast cells were consistent with the results of CIBERSORT and xCell. The presence of TNF-β family members, m6A and exosomes varied among the clusters. The analyses above confirmed that immune infiltration differed greatly in many respects among the clusters.

### Molecular biological states and oncogenic processes in ESCC

**Figure 5:**
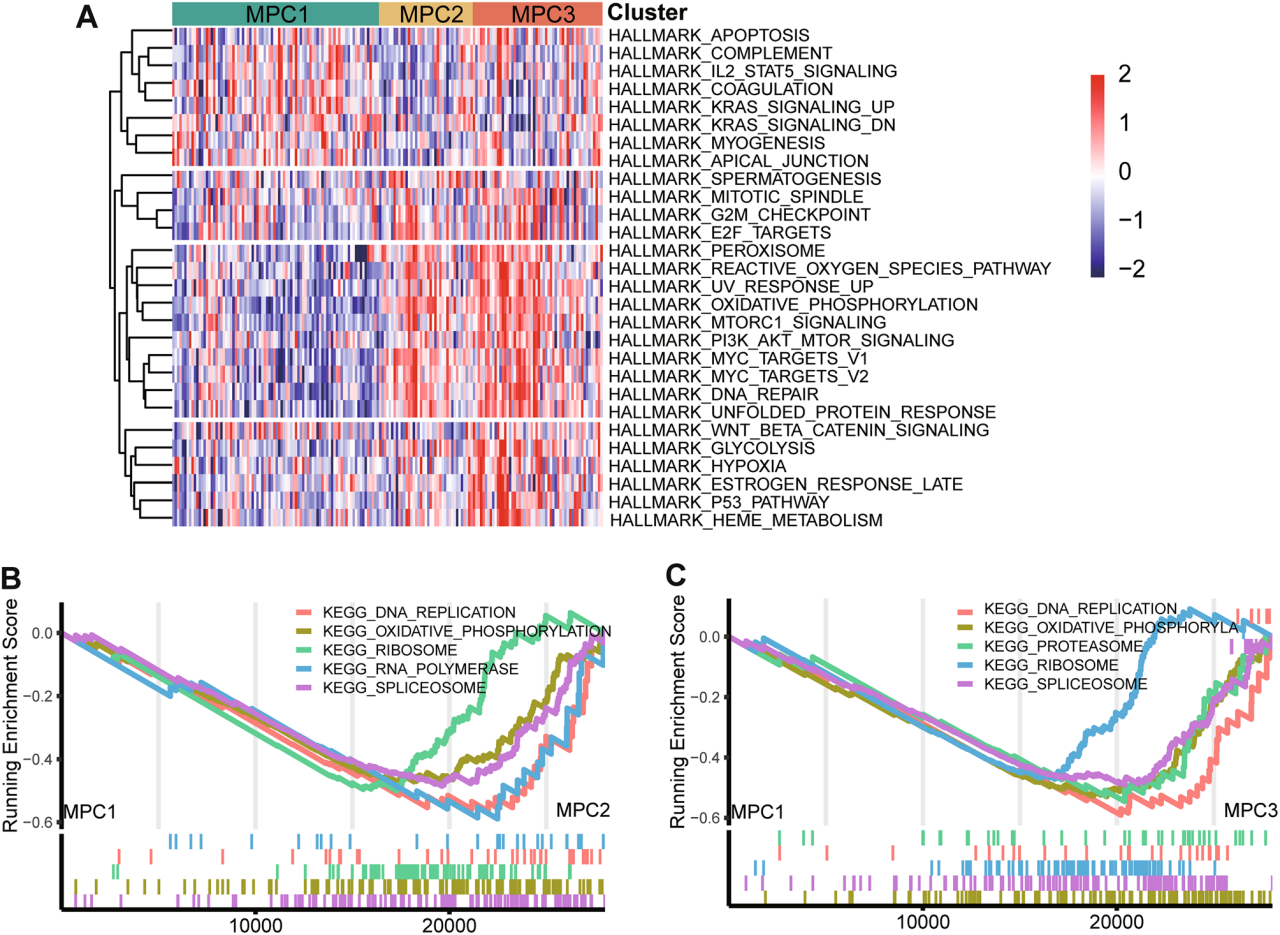
The biological pathways involved across metabolic pathway-based clusters in ESCC. (**A**) Heatmap showing normalized enrichment scores of hallmark pathways significantly differentiated among the three clusters. GSEA plot showing significantly upregulated and downregulated pathways with KEGG pathways in MPC1 versus MPC2 (**B**) and MPC1 versus MPC3 (**C**).

Several tumor hallmarks, such as mTORC1, PI3K-AKT-mTOR and KRAS, were activated in MPC3, and other tumor biological processes, including hypoxia, peroxisomes, MYC targets and oxidative phosphorylation, were upregulated in MPC3 (Fig. 5A). Similar significant hallmark gene sets (e.g., mTORC1 and the process of oxidative phosphorylation) were also found in the TCGA dataset (Fig. S6A).

We utilized the DEGs among the MPCs ranked by log2-fold changes to perform GSEA of the KEGG pathways. Compared to those of MPC1, MPC2 and MPC3 were significantly enriched in gene transcription elements such as ribosomes, spliceosomes, proteasomes and polymerases (Fig. 5B,C). Moreover, the activities related to the biological processes of cell growth and proliferation, such as DNA replication and oxidative phosphorylation, was markedly lower in MPC1. According to the TCGA cohort, the differences in MPC2 and MPC3 were apparent, and ribosomes were still meaningful between the two clusters (Fig. S6B–D).

### Comparison of metabolite levels among MPCs

**Figure 6:**
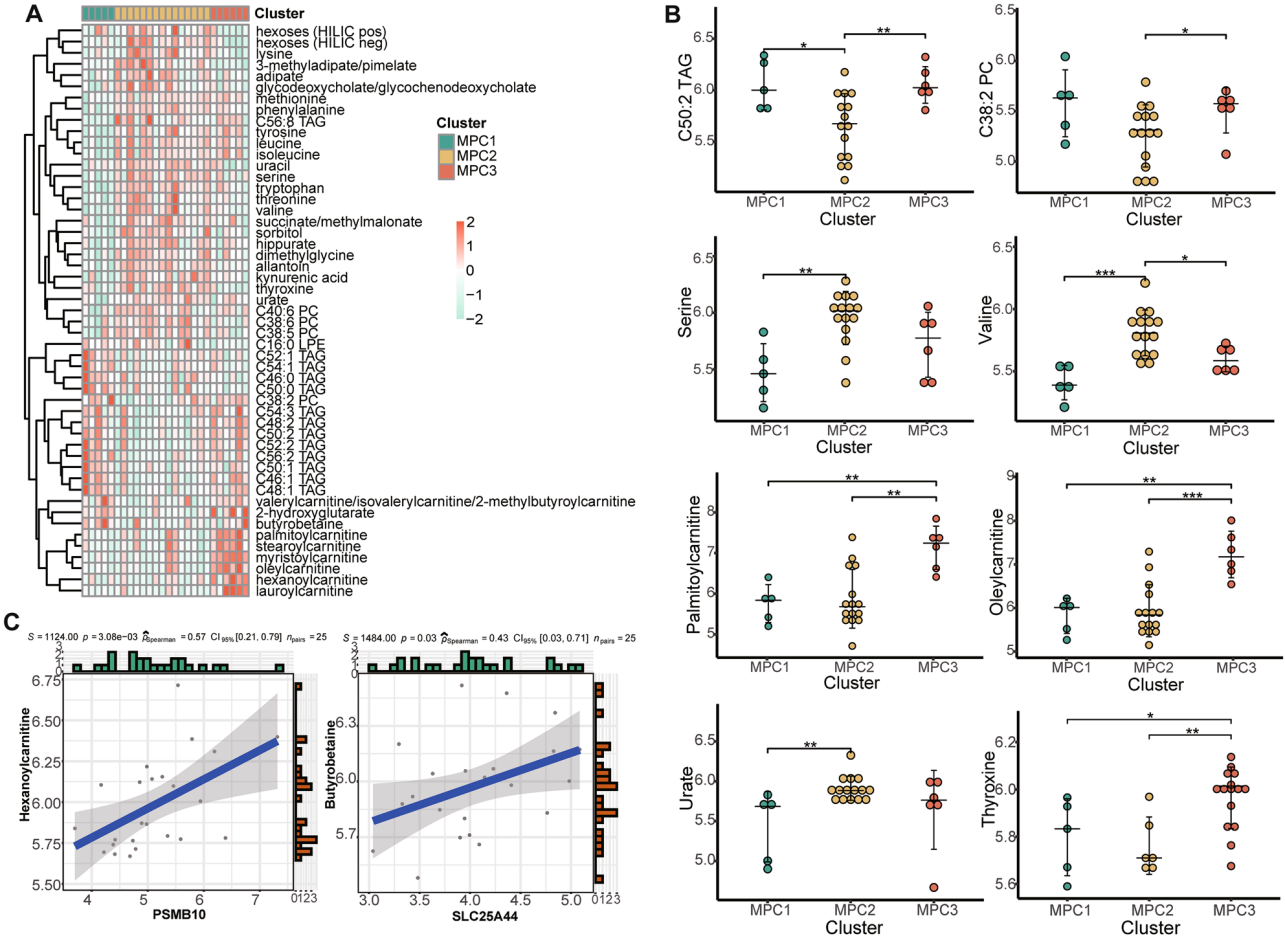
Metabolite profiles among metabolic pathway-based clusters. (**A**) Heatmap showing the relative intensity levels of metabolites significantly different among the three clusters. (**B**) Distribution of metabolite relative levels of lipids, amino acids, carnitine and other representative metabolites (urate and thyroxine). (**C**) Correlation diagram illustrating the relationship between hexanoylcarnitine and PSMB10 and between butyrobetaine and SLC25A44. * means *P* < 0.05; ** means *P* < 0.01; *** means *P* < 0.001.

Metabolic reprogramming can accompany alterations in intracellular and extracellular metabolites^6^. We employed an identical approach to classify the 26 human ESCC cell lines from the CCLE database. The cophenetic count in the NMF rank survey also revealed that the most common cluster rank number was three (Fig. S7A). We then categorized the cell lines into three clusters and further analyzed the differences in metabolite profiles between the clusters. A large number of amino acid metabolites were enriched in MPC2. Other functional substances, such as urate, were also abundant in MPC2. MPC1 contained a large quantity of lipids, mainly triacylglycerol (TAG), which was consistent with the aforementioned stratification. MPC3 was enriched in carnitine, associated with energy metabolism (Fig. 6A). We extracted significant metabolites across clusters and summarized the characteristics according to the relative intensity levels. The metabolite discrimination among MPCs was also noteworthy (Fig. 6B). MPC2 exhibited higher consumption of valine compared to MPC3. The major form of cellular signaling involves the reversible phosphorylation of proteins at tyrosine, serine, or threonine residues^28^, and our analysis revealed such trails in MPC2. The levels of palmitoylcarnitine and oleylcarnitine were the highest in MPC3, which was significantly different between the other two clusters. Carnitine palmitoyltransferase I might be a suppressive factor and emerging therapeutic target in cancer treatment. We also examined the levels of a representative lipid, C50:2 TAG, which were significantly different between MPC1 and MPC2. Meanwhile, the cell line expression levels of C38:2 PC (phosphatidylcholine) was also upregulated in MPC1 and MPC3. Furthermore, we examined the seven prognostic genes from the PPI network in the context of metabolites. Notably, there were positive correlations between carnitine abundance in MPC3 cells and the abundance of certain risk genes, specifically between hexanoylcarnitine and PSMB10, as well as between butyrobetaine and SLC25A44 (Fig. 6C). In essence, our study delved into the metabolite profiles of ESCC cell lines and contrasted the metabolite abundances across clusters.

### Drug sensitivity analysis of MPCs

**Figure 7:**
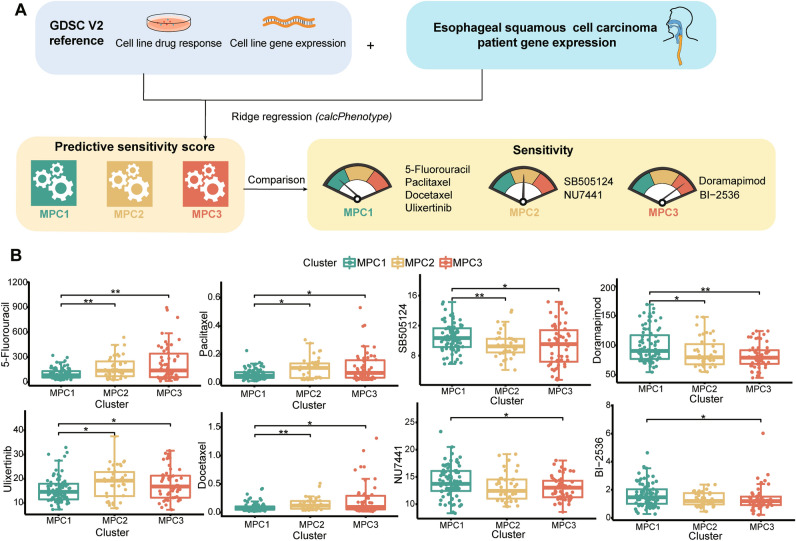
Drug sensitivity comparison across metabolic pathway-based clusters in ESCC patients (**A**) Workflow of the drug sensitivity prediction procedure. (**B**) Distribution of the estimated drug sensitivities of 5-fluorouracil, paclitaxel, docetaxel, ulixertinib, SBS05124, NU7441, doramapimod and BI-2536. * means* P* < 0.05; ** means *P* < 0.01.

We also utilized transcriptome data to predict potential drug sensitivity against the GDSC database. The sensitivity scores of each drug were quantified for each sample and compared among the three MPCs (Fig. 7A). We found that 57 drugs, including five commonly used chemotherapy drugs, exhibited statistically significant differences among the three MPCs. Specifically, the sensitivity scores to 5-fluorouracil (an inhibitor of DNA and RNA synthesis), paclitaxel (a microtubule disassembly inhibitor), docetaxel (a microtubule disassembly inhibitor), and ulixertinib (a MEK1/2 inhibitor) were significantly lower for MPC1, while doramapimod (a p38 MAPK inhibitor) had the lowest sensitivity score for MPC3 (Fig. 7B). Additionally, SB505124 (a TGF-β signaling pathway inhibitor) and NU7441 (a DNA-PK inhibitor) were more sensitive to MPC2, while BI-2536 (a PLK1 inhibitor) was also sensitive to MPC3.

### Associations between MPC subtyping and immune response

**Figure 8:**
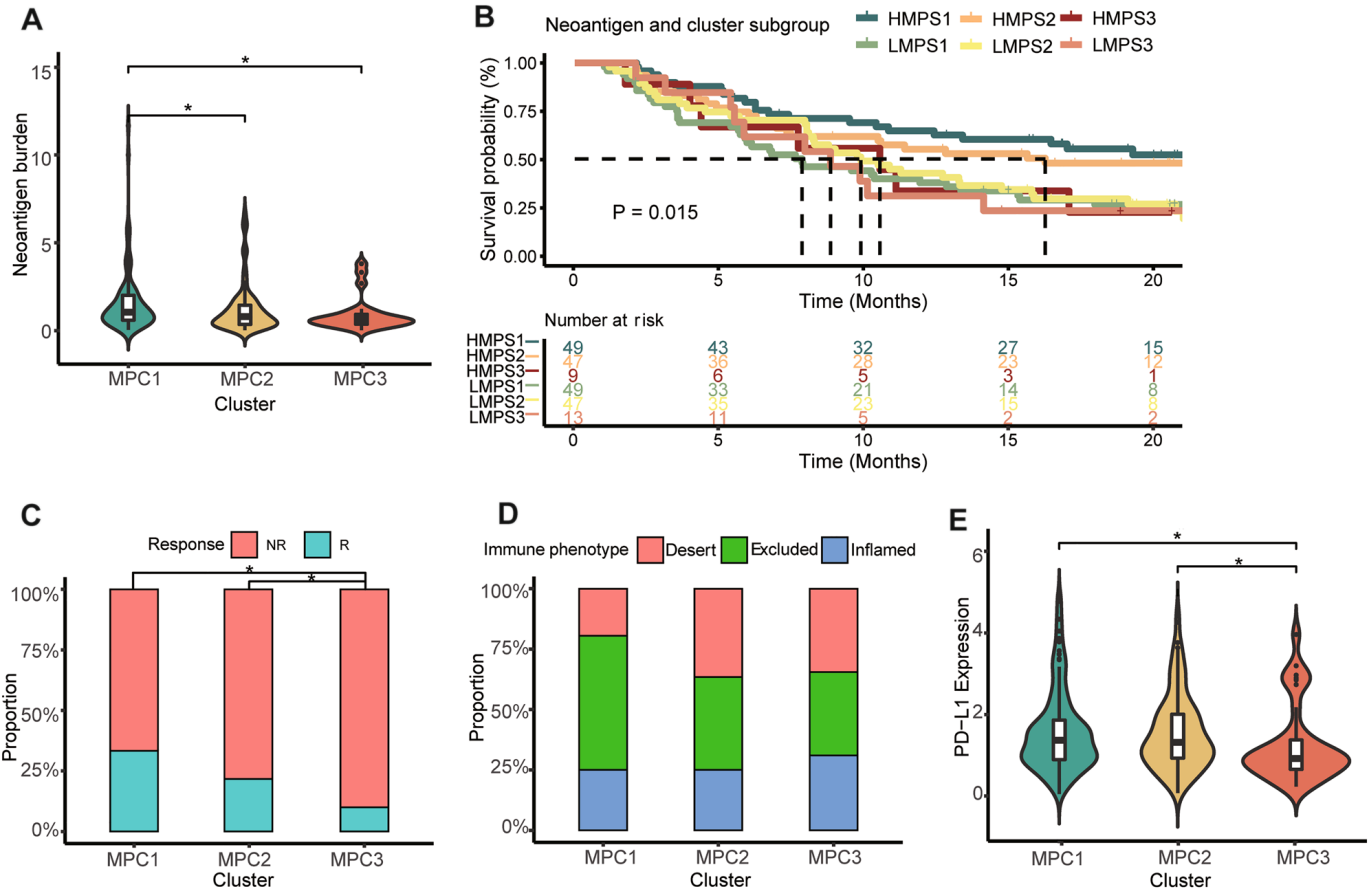
Immune response characteristics of patients in the anti-PD-L1 cohort with MPCs in ESCC. (**A**) Violin plot illustrating the tumor neoantigen burden in samples across MPCs. (**B**) K‒M curves of OS in patients according to the integrated neoantigen burden and cluster subgroup. Bar plot showing the proportions of patients with an immune response (**C**) and immune phenotype (**D**) in the three clusters. (**E**) Violin plot demonstrating PD-L1 expression in samples across MPCs. * means *P* < 0.05.

The metabolic profiles of an independent anti-PD-L1 immunotherapy cohort derived from bladder cancer tissue (also squamous carcinoma tissue) were also classified into three clusters with NMF based on RNA-seq data. These results might provide insight into the immunotherapy efficacy of MPCs in ESCC. The NMF rank survey indices were also plotted (Fig. S7B). Tumors in MPC1 had a high neoantigen burden (Fig. 8A), which predicted a better therapeutic response to immune checkpoint inhibitors^29^. The Kaplan‒Meier curve and TMB landscape are shown in Fig. S7C and D. We combined clusters and neoantigen burden statuses to establish an elaborate classification system for survival analysis and found significant differences among the subgroups (*P* = 0.015; Fig. 8B). We found that the survival outcomes varied greatly among patients with different MPC1 subtypes. HMPC1 had a far better OS than LMPC1. These clinical findings indicated that patients with a high abundance of the neoantigen burden in MPC1 might have a favorable immune response that is beneficial for survival. Moreover, compared with MPC1 and MPC2, MPC3 had the worst immune response (*P* < 0.001, Fig. 8C). The immune-excluded type accounted for a large proportion of the MPC1 subgroup, and the immune-inflamed type accounted for a high proportion of the MPC3 subgroup (Fig. 8D). These results confirmed our findings that the cancer cells harboring MPC1 had high PD-L1 expression (Fig. 8E).

## Discussion

Advances in diagnostic or therapeutic techniques are accompanied by the discovery of new markers or classifications^30^. In ESCC, previous studies have investigated the molecular subtypes of m6A RNA methylation modulator expression and mutated genes and revealed mutational signatures, prognostic proteins, and cell-in-cell (CIC) structure profiles^12,13,31^. Metabolic heterogeneity classification can also help to interpret the variations in ESCC patients^32^, and it has been studied in several other types of cancer^33–35^. These studies also focused on several metabolites, such as palmitic acid (PA), tyrosine and metabolite complexes, and studied the downstream mechanism of these metabolites and the prediction of patient prognosis in ESCC^36,37^. However, previous classifications of gene expression were based on conventional transcriptomic data. In our study, we processed the transcriptomics data to obtain enrichment scores for metabolic pathways. The metabolic pathways used for classification could clearly express the metabolic characteristics of ESCC. In addition, previous studies have rarely addressed metabolic classification in ESCC, and our stratification might provide new implications for targeted therapy.

Tumor tissues not only require nutrient uptake but also demand specific ways for use^6^. We found that the energetic cluster (MPC3) was correlated with worse disease outcomes in ESCC patients. The characteristic high glycolytic expression of MPC3 was also identified in another hallmark gene set. Tumor tissues require high energy consumption to meet the needs of cell growth and proliferation. Upregulated glycolytic activities could adapt to these features and supply additional energy^38^. Cofactor metabolism in cancer could enable NADPH to participate in reductive biosynthesis and redox homeostasis, and upregulated de novo nucleotide metabolism enables cells to proliferate rapidly^39,40^. In addition, the metabolic intermediates of glycolysis play a crucial role in macromolecular biosynthesis. These compounds can promote the generation of NADPH and ribose-5-phosphate. The production of NADPH enables cancer cells to maintain reduced levels of glutathione (GSH), which might protect tumor cells against antineoplastic and chemotherapeutic agents^41^. In addition, Peng et al. reported that upregulated vitamin and cofactor metabolism was associated with poor prognosis in a cohort of 9,125 TCGA samples across 33 cancer types^42^.

The DEGs identified from the PPI network were related to metabolic heterogeneity and protein expression in ESCC tissues. The core gene E2F1 regulates metabolism in cancer cells by enhancing glycolysis and has been shown to regulate oxidative metabolic genes in muscle and fat. These functions can contribute to the reprogramming of energy metabolism in tumor cells, which is required for proliferation and cancer progression under hypoxic and nutrient-deprived conditions^43^. We also found that the lncRNAs TPM1-AS and LINC01132 were highly expressed in MPC3. TPM1-AS is a natural antisense lncRNA that plays a crucial role in regulating the alternative splicing of TPM1. Moreover, TPM1-AS is also implicated in TPM1-mediated filopodium formation and the migration of cancer cells^44^. A previous study also demonstrated that LINC01132 overexpression promoted cell growth, proliferation, invasion and metastasis in vitro and in vivo^45^. Loss of the P53 gene may lead to dysregulation of NER, which is an important and necessary event in the pathogenesis of BRCA1-mutated tumors^46^. BRCA1 was also identified as a prognostic gene in the PPI network. ZNF750 is a novel significantly mutated gene in ESCC that is significantly mutated in MPC3. It may act as a tumor suppressor by directly repressing SNAI1 (an oncogene) and inhibiting the epithelial–mesenchymal transition (EMT) process in ESCC and other types of SCC^47^. Additionally, ZNF750 exhibited comutational associations with several cilia function-related genes, namely, DNAH3 and HYDIN, meriting further exploration. Moreover, MPC3 had a greater mutational burden, which might be associated with poor OS. Liu et al. investigated whether patients with a lower TMB had longer OS in some cancer types^48^. In terms of the extracted mutational signatures, a previous study revealed that the APOBEC cytidine deaminase signature was significant in 14 other cancer types. This finding might also reveal one of the probable processes of carcinogenesis in ESCC. This process converts cytosine to uracil during RNA editing and retrovirus or retrotransposon restriction^49^. The COMSIC SBS16 signature, which is predominantly enriched in MPC3, has been found to be associated with alcohol consumption^50^. This distinct pattern is typically associated with EC, suggesting that MPC3 might more accurately reflect the mutational landscape of ESCC^51^.

We also found upregulation of resting mast cells and regulatory T cells (Tregs) and downregulation of CD8+ T cells in the MPC3 population. A higher proportion of CD8+ T cells was associated with better patient outcomes, whereas a high mast cell density was related to the progression of ESCC and poor disease outcome^13,52^. A high concentration of Tregs in ESCC can lead to immune escape and promote tumor progression^53^. Several common oncogenic pathways, such as the MTORC1, PI3K-AKT-MTOR and KRAS signaling pathways, as well as pathways related to hypoxia status, MYC targets and oxidative phosphorylation, were enriched in MPC3. The biological processes or statuses verified the aggressiveness of MPC3.

The MPC2 cell lines exhibited increased uptake of amino acids, including serine and valine. Amino acid derivatives contribute to epigenetic regulation and immune responses linked to tumorigenesis and metastasis^54^. Other metabolites, such as thyroxin and urate, were also highly consumed by the MPC3 cell lines. Patients with higher serum uric acid (SUA) levels may have an unfavorable survival probability, and numerous studies suggest that triiodothyronine and thyroxin have cancer-stimulating effects^55,56^. MPC3 was characterized by high expression of acylcarnitines (palmitoylcarnitine, oleylcarnitine, etc.). The cofactor carnitine allows fatty acid acyl moieties to enter the mitochondrial matrix, where these molecules are oxidized via the β-oxidation pathway^57^. Mitochondria are the powerhouses of cells and provide energy, greatly affecting lifespan. TAGs were found to be significantly highly expressed in MPC1. Fatty acids (FAs) are implicated in the synthesis of phospholipids and TAGs. A mixed outcome of upregulation and downregulation of saturated and unsaturated FAs in both tissue and serum was demonstrated. UFAs are related to a reduced risk of ESCC^58^. Furthermore, SLC25A44 expression was positively associated with carnitine levels. SLC25A44 mediates the transport of branched-chain amino acids into mitochondria, which helps control energy homeostasis^59^.

Several drugs (paclitaxel, platinum, 5-fluorouracil, capecitabine, tegafur, etc.) are commonly used for ESCC chemotherapy. The commonly utilized antitumor drugs demonstrated marked sensitivity to MPC1, as evidenced by the low scores. In contrast, doramapimod exhibited elevated sensitivity, specifically in the context of MPC3. Doramapimod is an anti-inflammatory compound that acts through p38 MAPK inhibition and is a potent inhibitor of TNF-α and IL-1β to combat inflammation, showing great potential in MPC3 therapy^60^. In addition, MPC3 had the worst immune response, and MPC1 might be sensitive to anti-PD-L1 drugs, which could provide clues for precision therapy. The patients in whom MPC1 had a low neoantigen burden had a worse OS than did the other patients, which also indicates the great clinical significance of anti-PD-L1 therapy for MPC1.

Although we have made significant progress in understanding the metabolic heterogeneity of ESCC, there are a few limitations in our study. First, the tumor samples included in our study were retrieved from retrospective datasets, and a prospective cohort of ESCC patients is needed to validate our findings. Second, it is necessary to further expand the sample size to explore the mutational and hallmark profiles of ESCC in future research. Third, in the present study, the anti-PD-L1 immunotherapy cohort was collected from patients with squamous cell carcinoma of the bladder, and the potential role of MPCs in guiding immunotherapy was revealed. Finally, our conclusions are based on the results of bioinformatic analysis of datasets, which need to be further verified in clinical studies.

Overall, metabolic heterogeneity was analyzed by analyzing transcriptome data enriched in metabolic pathways in ESCC patients. We divided the ESCC patients into three metabolically characterized clusters. The three clusters showed distinct features in terms of immune status, mutation status, prognosis, and metabolites. The main characteristics of the MPCs are summarized in Table S5. These differences could help us make more precise clinical decisions for therapy.

### Supplementary Information

Supplementary Figures.

Supplementary Tables.

## Data Availability

The sources of all the data used in this research are included in this article.
