# Supplementary material for: Genetic and molecular characterization of metabolic pathway-based clusters in esophageal squamous cell carcinoma
Source: Sci Rep. 2024 Mar 14;14:6200. doi: 10.1038/s41598-024-56391-w (PMC10940668; doi:10.1038/s41598-024-56391-w)
Supplement: Supplementary file 1 — Supplementary Figures. [file 41598_2024_56391_MOESM1_ESM.docx]

**Supplementary figures**

**
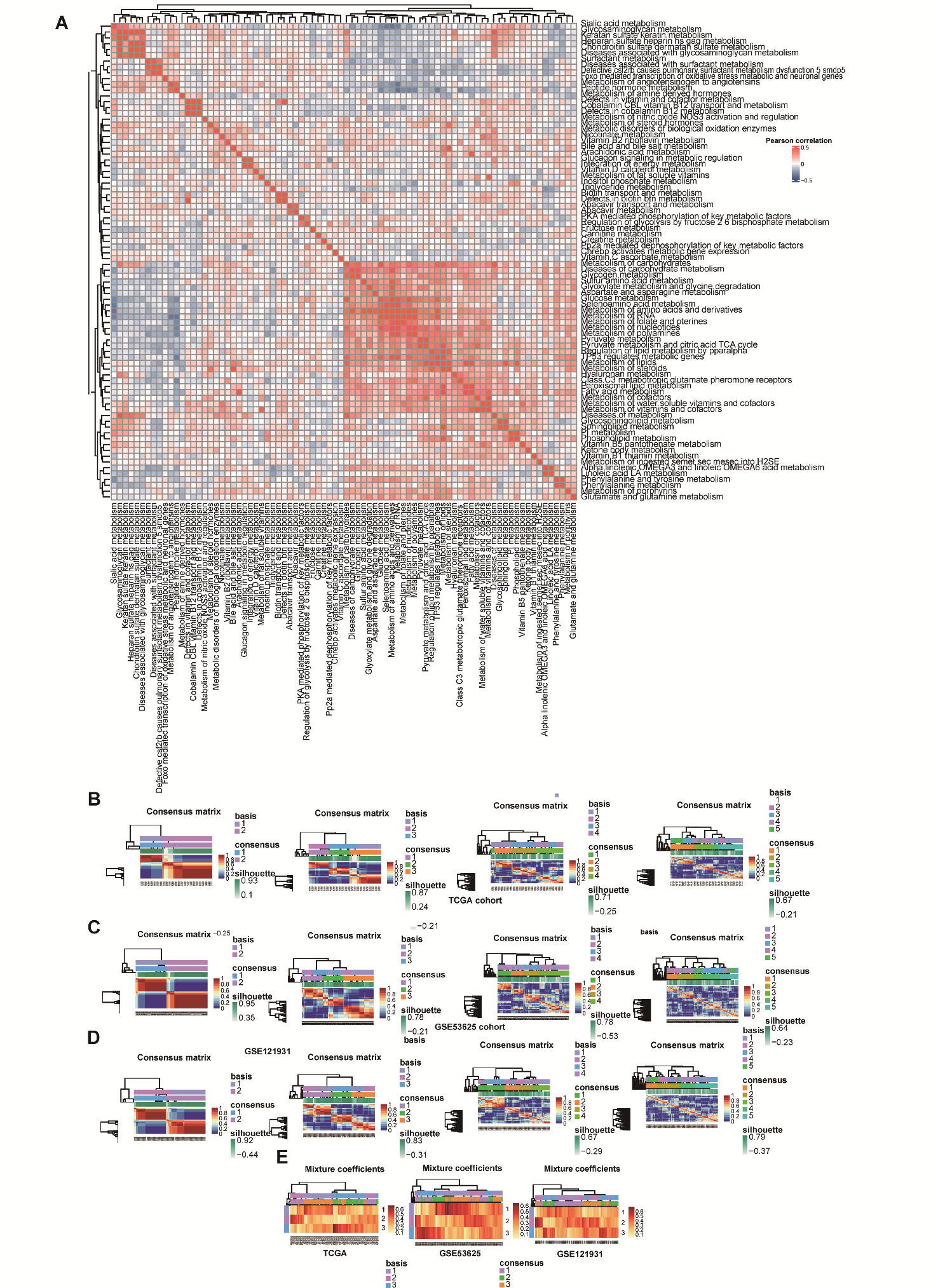
**

**Supplementary figure 1. Exploration of the** **stratification of metabolic-pathway-based clusters.** (A) The correlations among metabolic pathways were analyzed by Pearson correlation. Consensus matrix of metabolic-pathway-based stratification from rank two to five using NMF method in TCGA (B) and GSE53625 (C) and GSE121931 (D) cohorts. (E) Heatmap showing the mixture coefficients of rank three classification in the three datasets.

**
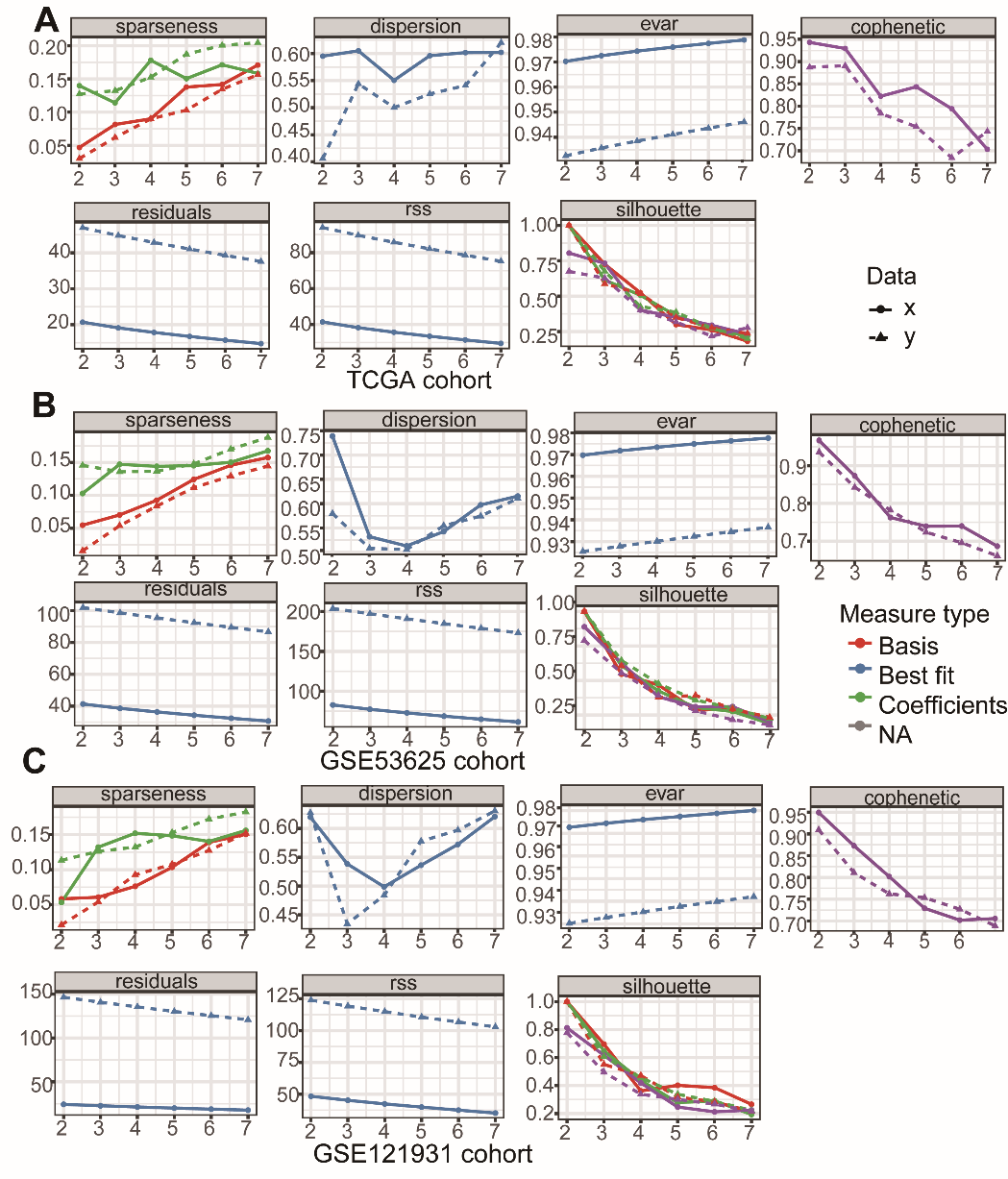
**

**Supplementary Figure 2. NMF rank survey indices of metabolic-pathway-based stratification in TCGA (A) and GSE53625 (B) and GSE121931 (C) cohorts.**

**
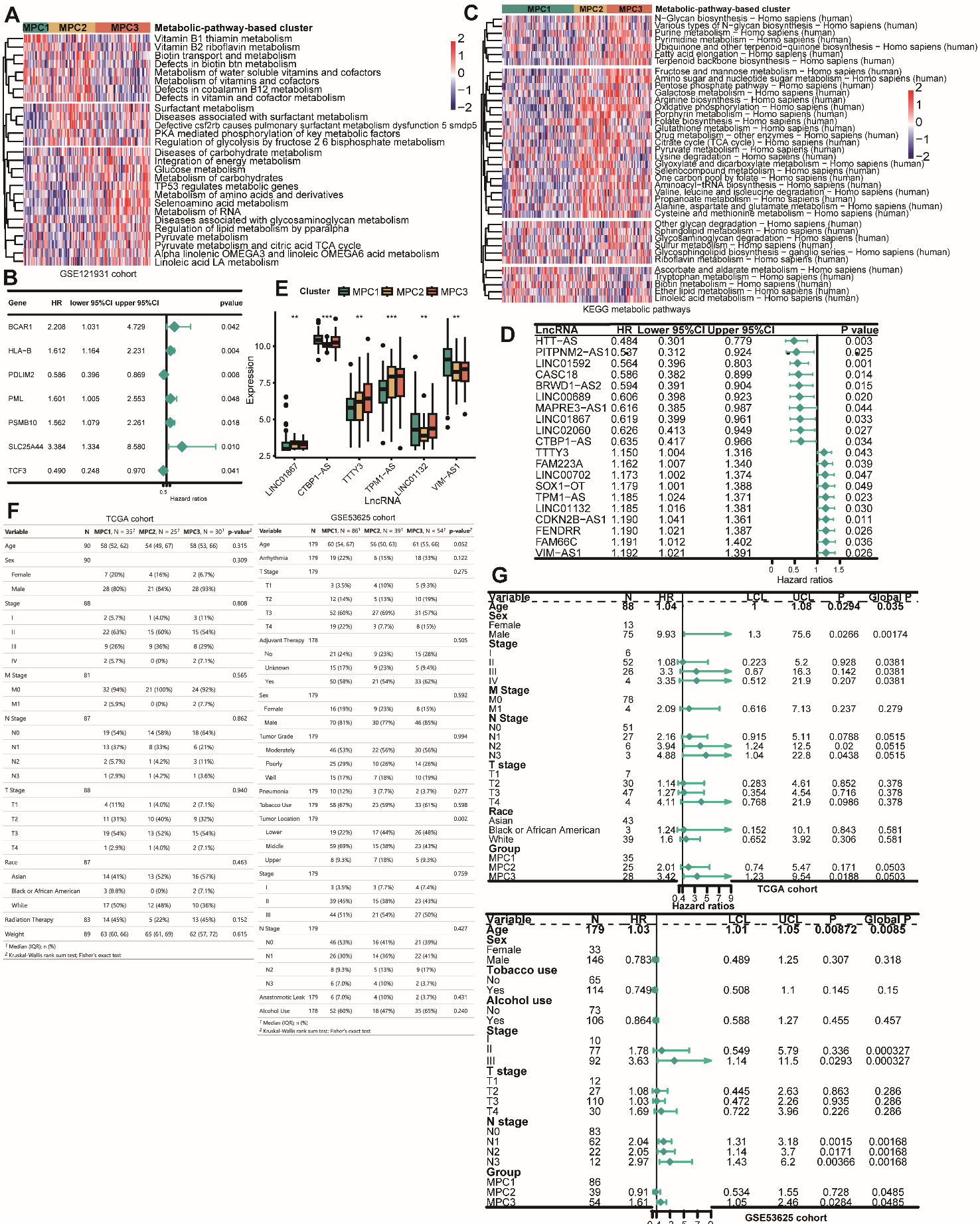
**

**Supplementary Figure 3. Metabolic-pathway-based stratification validation and prognostic information.** (A) Heatmap showing normalized enrichment scores of the three MPCs in GSE121931 cohort. (B) Heatmap showing normalized enrichment scores of the three MPCs with KEGG metabolic pathways in GSE53625 cohort. (C) Forest plot of PPI genes correlated with prognosis using Univariate Cox regression. (D) Forest plot of top 20 hazard ratio (HR) lncRNAs positively or negatively correlated with prognosis using Univariate Cox regression. (E) Distribution of top prognostic lncRNAs with expression differences among MPCs. (F) Baseline characteristics of patients in the TCGA and GSE53625 datasets. (G) Forest plot of hazard ratio (HR) with clinical characteristics using Univariate Cox regression.

**
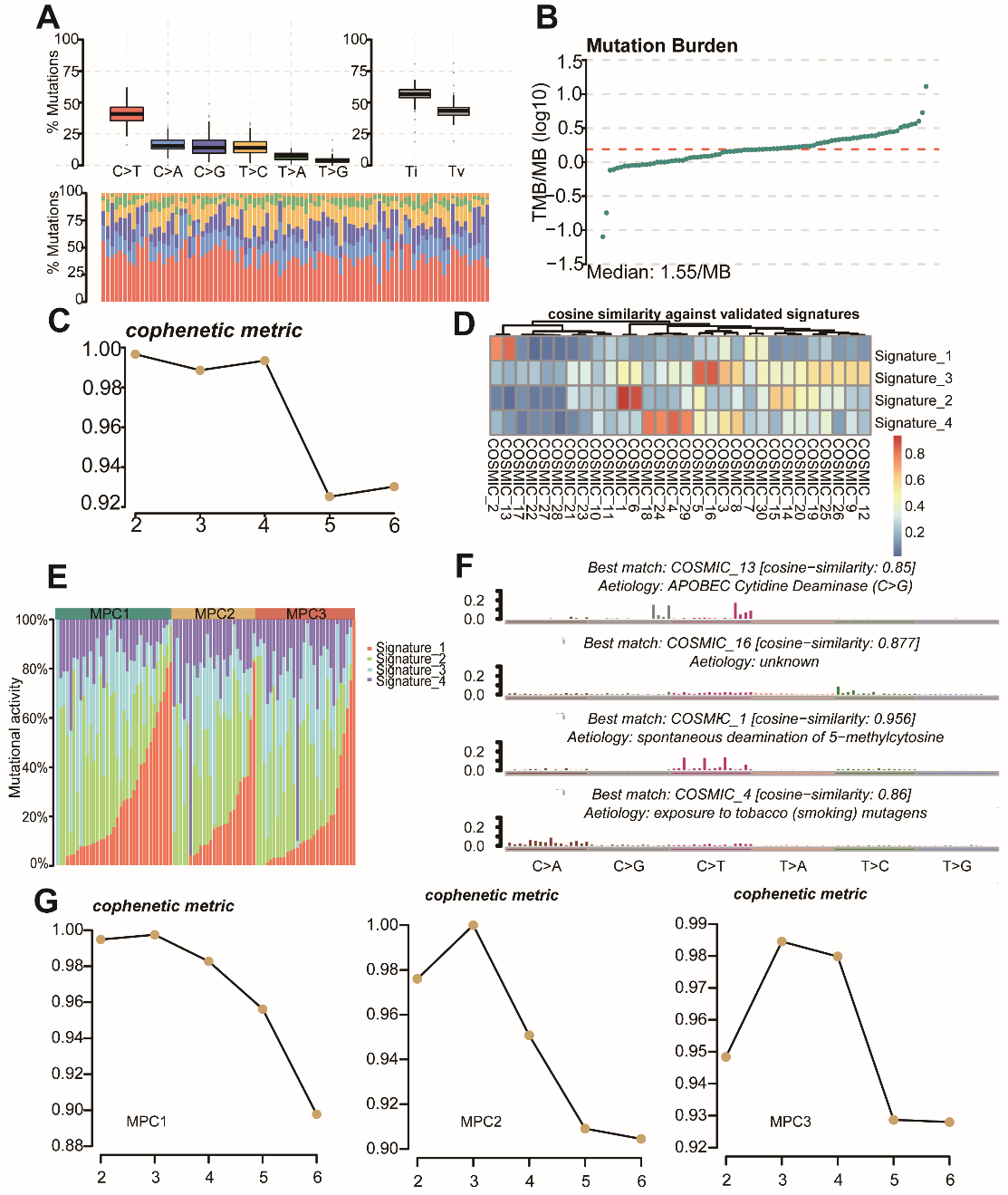
**

**Supplementary Figure 4. Mutational profiles in ESCC in TCGA cohort.** (A) Base substitution (transition and transversion) landscape in ESCC. (B) Dot plot showing the tumor mutation burdens across samples. (C) Line chart showing the exploration of cophenetic metric of extracted signatures in ESCC. (D) Heatmap showing cosine similarity against validated signatures of extracted signatures in ESCC. (E) The distribution of extracted mutational signatures in the three clusters. (F) The relationship of clinical and molecular characteristics with curated mutational signatures in ESCC. (G) Line chart showing the exploration of cophenetic metric of extracted signatures in MPCs.

**
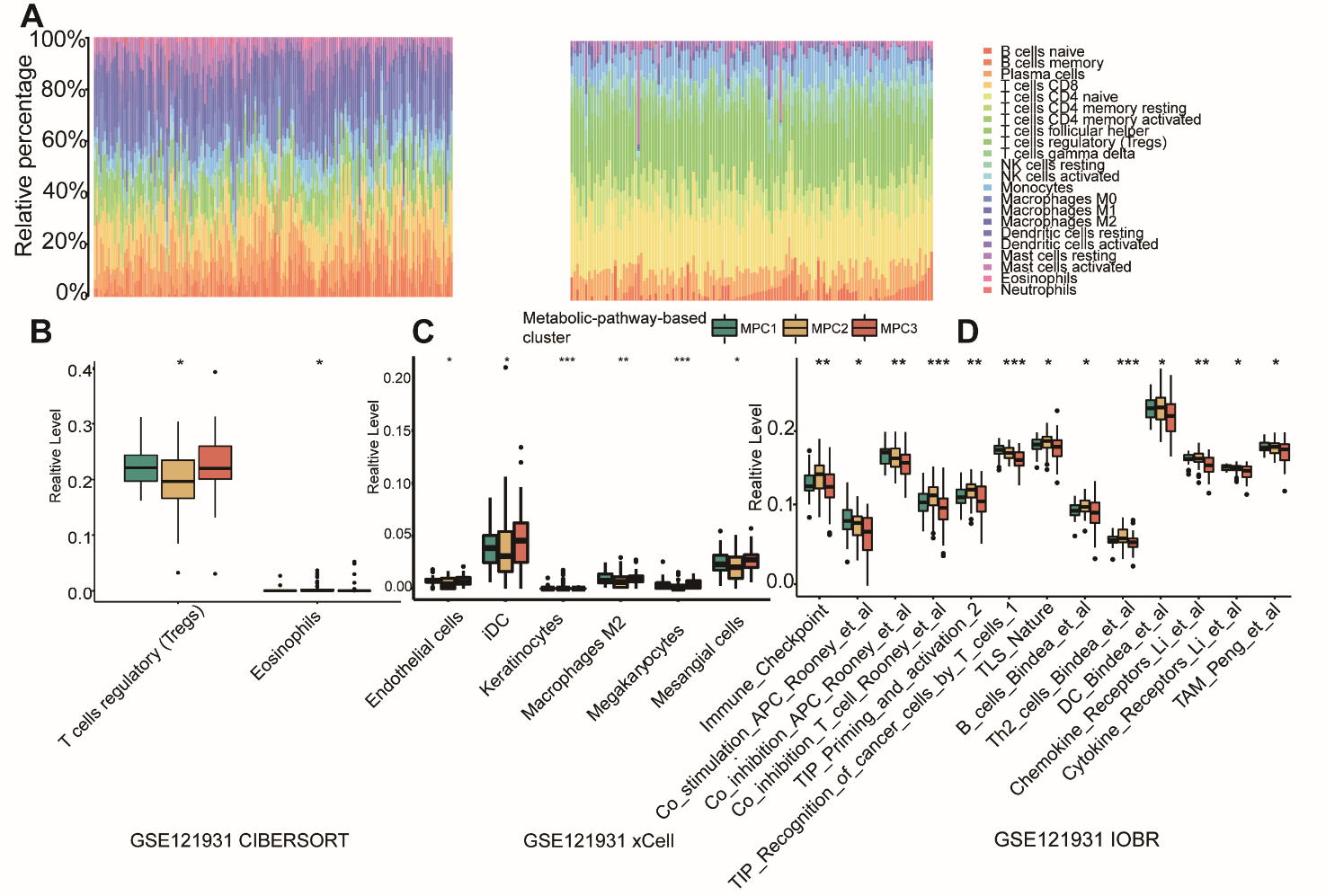
**

**Supplementary Figure 5. Immune infiltration profiles and validation across metabolic-pathway-based clusters in GSE121931 cohort.** (A) Bar plot showing the proportion of infiltrated immune cells calculated by the CIBERSORT algorithm in GSE53625 and GSE121931 dataset. The box plot showing the difference between infiltrated immune cells and signatures in TME with CIBERSORT (B), xCell (C) and IOBR (D) algorithm.

**
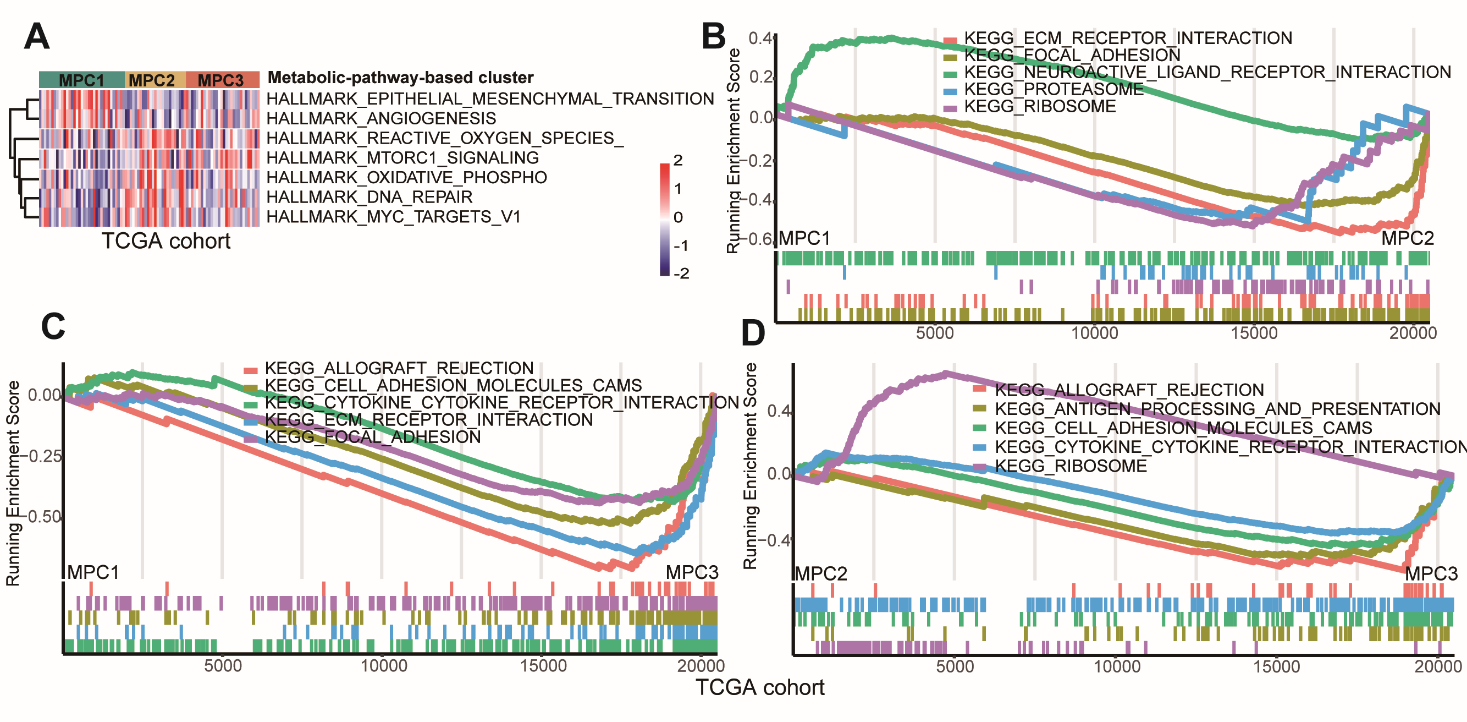
**

**Supplementary Figure 6. The validation of biological pathways involved across metabolic-pathway-based clusters in ESCC in TCGA cohort.** (A) Heatmap showing normalized enrichment scores of hallmark pathways statistically differentiated among the three clusters. GSEA plot showing significant upregulated and downregulated pathways with KEGG pathways in MPC1 versus MPC2 (B), MPC1 versus MPC3 (C) and MPC2 versus MPC3 (D).

**
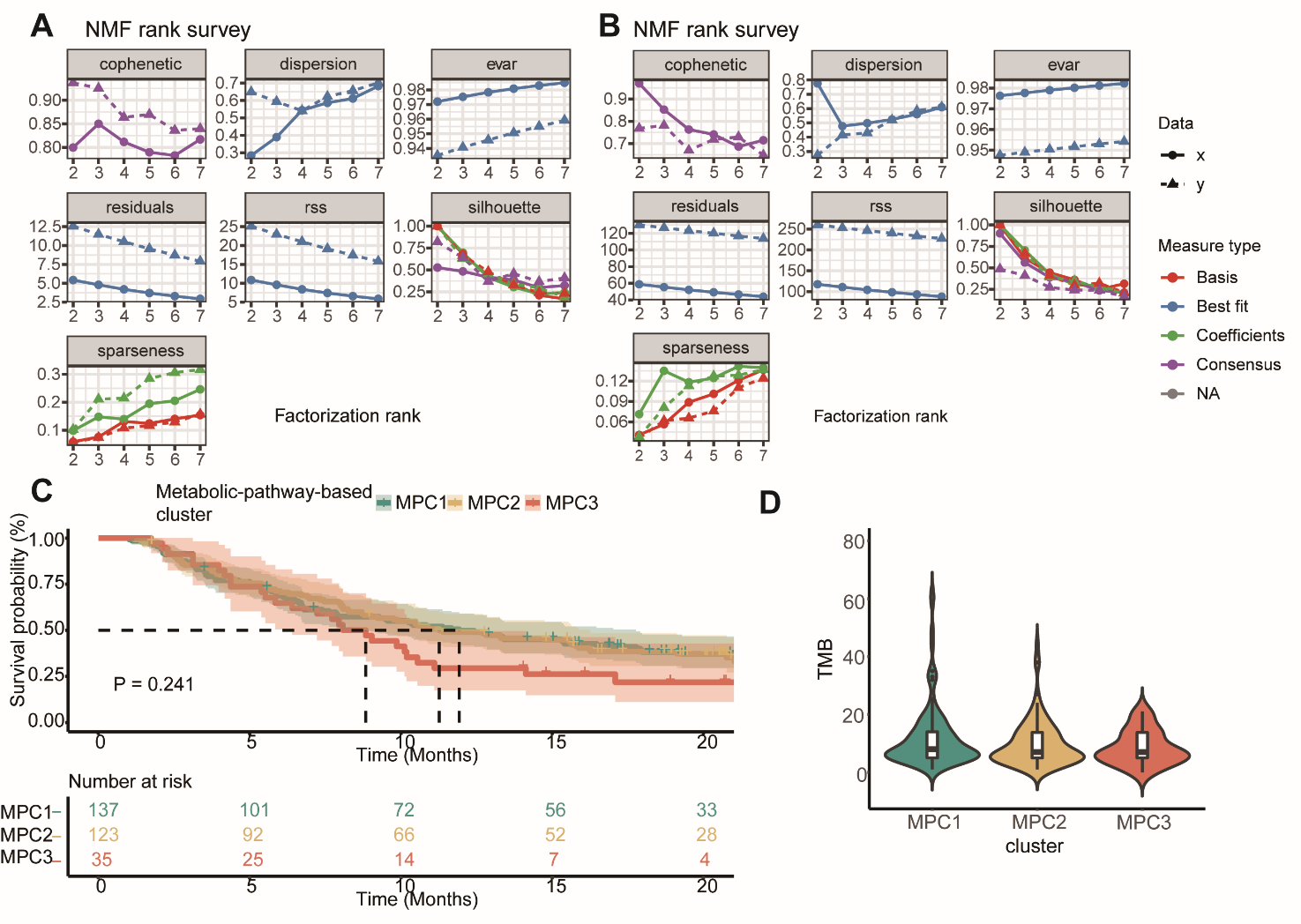
**

**Supplementary Figure 7. NMF stratification in ESCC cell lines and anti-PD-L1 cohort with clinical feature across metabolic-pathway-based clusters.** NMF rank survey indices using in stratification of ESCC cell lines (A) and immune response cohort (B). (C) Kaplan-Meier curves of overall survival (OS) among the three MPCs in immune response cohort. (D) Violin plot showing tumor mutation burden in samples across MPCs in immune response cohort.
